# Upregulation of ZHX2 predicts poor prognosis and is correlated with immune infiltration in gastric cancer

**DOI:** 10.1002/2211-5463.13160

**Published:** 2021-05-24

**Authors:** Anqi Cheng, Xiong Guo, Xinglong Dai, Ziwei Wang

**Affiliations:** ^1^ Department of Gastrointestinal Surgery Laboratory Research Center The First Affiliated Hospital of Chongqing Medical University China

**Keywords:** gastric cancer, immune infiltration, prognosis, ZHX2

## Abstract

The transcriptional repressor zinc finger homeobox 2 (ZHX2) is reported to regulate tumor progression in several human cancers, although little is known about its role in gastric cancer (GC). In the present study, we investigated the expression of ZHX2 and its relationship with the clinicopathological characteristics and prognosis of GC patients, and we also examined the effect of ZHX2 overexpression in GC cell lines. We used UALCAN (http://ualcan.path.uab.edu) and the Tumor Immune Estimation Resource (http://cistrome.org/TIMER) to examine ZHX2 mRNA expression, and also used Kaplan–Meier Plotter (https://kmplot.com) to determine whether ZHX2 expression was related to GC prognosis. Expression of ZHX2 protein was detected using immunohistochemical staining assays. Cell proliferation was evaluated using a cell counting kit‐8 and colony formation assays, whereas apoptosis was examined by flow cytometry. Wound healing and transwell assays were used to detect cell migration and invasion. We also performed Gene Set Enrichment Analysis (https://www.gsea‐msigdb.org) and used The Cancer Genome Atlas database (https://www.genome.gov/Funded‐Programs‐Projects/Cancer‐Genome‐Atlas) to examine the correlation of ZHX2 with immune infiltration. We report that ZHX2 is highly expressed in GC tissues and is significantly associated with clinical characteristics. Upregulation of ZHX2 predicted poor prognosis in GC. Furthermore, ZHX2 overexpression can promote the proliferation, invasion and migration, but inhibit apoptosis, of GC cells. High expression of ZHX2 in GC is correlated with the presence of infiltrating immune cells, including B cells, CD4^+^ T cells, macrophages and dendritic cells. Our data suggest that high expression of ZHX2 in GC predicts poor prognosis. In addition, ZHX2 may promote malignant behaviors of GC cells, and immune infiltration might be related to the oncogenic role of ZHX2 in GC.

## Graphical abstract

ZHX2 is upregulated in gastric cancer tissues at both the mRNA and protein level as validated by bioinformatic analysis and immunohistochemistry; moreover, its expression is correlated with the prognosis of gastric cancer patients. Upregulation of ZHX2 may promote malignance of gastric cancer cells. Furthermore, immune infiltration might underlie the oncogenic role of ZHX2 in gastric cancer.

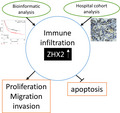

**Abbreviations**
CCK‐8: cell counting kit‐8
DFS: disease‐free survival
FP: first progression
GC: gastric cancer
GSEA: Gene Set Enrichment Analysis
HD: homeodomains
IHC: immunohistochemical
OS: overall survival
PPS: post progression survival
qRT‐PCR: quantitative real‐time quantitative PCR
TCGA: The Cancer Genome Atlas
TIMER: Tumor Immune Estimation Resource
ZHX2: zinc finger homeobox 2

Gastric cancer (GC) ranks as the fifth most common cancer in the world, with approximately 1 030 000 new cases and 783 000 deaths in 2018 [1]. As a result of the development of diagnostic technology and standardized clinical treatment, GC therapy has made much progress, and surgery combined with chemotherapy has become the standard treatment for advanced GC in most countries [2, 3]. However, because of a lack of early typical symptoms, most patients are diagnosed at advanced stages. In addition, the total GC survival rate is still discouraging, and the 5‐year overall survival (OS) rate is lower than 40% [4, 5]. The molecular mechanisms of GC tumorigenesis remain to be clearly clarified to provide novel diagnostic and treatment options.

The zinc fingers and homeoboxes family includes ZHX1, ZHX2 and ZHX3, which contain two zinc fingers and four or five homeodomains (HD). ZHX2, mainly located in the cell nucleus, is widely expressed in multiple human organs, including the heart [6], lung [7], kidney [8], and so on. ZHX2 was initially found as a negative regulation gene of AFP [9] and was subsequently demonstrated to have a tumor suppressive role in hepatocellular cancer [10]. Moreover, ZHX2 was demonstrated to be highly expressed in renal clear cell carcinoma and to activate the nuclear factor‐kappa B pathway via epigenetic modification to promote cancer cell proliferation and migration [11]. The expression of ZHX2 in different cancers shows heterogeneity and the role of ZHX2 among different cancers is conflicting. A recent study showed that ZHX2 was upregulated in GC tissues using online data [12], although the expression and biological function of ZHX2 in GC still remain to be elucidated.

In the present study, we explored the expression of ZHX2 and its relationship with the clinicopathological characteristics and prognosis of GC patients. Moreover, we investigated the role of ZHX2 with respect to the proliferation, apoptosis, migration and invasion of GC cells. Additionally, we revealed the relationship of ZHX2 with immune infiltration. The results obtained indicate that ZHX2 is highly expressed in GC and predicts poor survival. ZHX2 promotes the malignant properties of GC cells partially via interacting with immune infiltration cells.

## Materials and methods

### Tumor Immune Estimation Resource

Tumor Immune Estimation Resource (TIMER) (http://cistrome.org/TIMER) comprises a tool for inferring the correlation between ZHX2 and the abundance of tumor‐infiltrating immune cells using a deconvolution method. Immune infiltrate cells, including CD8^+^ cells, B cells, CD4^+^ cells, macrophages, neutrophils and dendritic cells, were analyzed separately. The left panel indicates the expression level of ZHX2 against tumor purity, whereas the *x*‐axis indicates the immune infiltration level. Additionally, the diff Exp module was applied to evaluate the differential expression of ZHX2 between tumor tissues and adjacent normal tissues in all tumors in The Cancer Genome Atlas database (https://www.genome.gov/Funded‐Programs‐Projects/Cancer‐Genome‐Atlas).

### UALCAN

UALCAN (http://ualcan.path.uab.edu) is an effective online tool facilitating data mining and the analysis of RNA‐sequencing data from TCGA databases. In the presdent study, we applied UCLCAN to analyze the expression of ZHX2 in tumor tissues and its correlation with clinicopathologic characteristics in GC.

### Kaplan–Meier Plotter

Kaplan–Meier Plotter (https://kmplot.com) is a practical tool with a powerful role with respect to analyzing the relationship between gene expression and prognosis in patients with cancer. Kaplan–Meier Plotter was performed to explore the correlation between the mRNA expression of ZHX2 and the survival of GC patients, including OS, first progression (FP) and post progression survival (PPS).

### Clinical samples

All 60 GC samples and corresponding adjacent normal gastric tissues were collected from GC patients at the First Affiliated Hospital of ChongQing Medical University from January 2014 to June 2015. The protocol for the study had been approved by the Ethics Committee of the First Affiliated Hospital of ChongQing Medical University. Each patient provided their written informed consent, and no patient received radiotherapy or chemotherapy before radical gastrectomy. Basic patient information was collected. All procedures were conducted in accordance with the Helsinki declaration.

### Immunohistochemistry

Immunohistochemical (IHC) staining was conducted to measure the protein expression level of ZHX2 in GC tissues and corresponding adjacent normal tissues. The surgically collected tissue samples were fixed in 4% paraformaldehyde and embedded. The slices were baked for 24 h at 60 °C, then deparaffinized in dimethylbenzene, rehydrated, and finally rinsed with phosphate‐buffered saline for 5 min, three times. Next, the slices were incubated with 3% hydrogen peroxide. For antigen retrieval, the slices were immersed in citric acid buffer and subsequently heated for 10 min at 95 °C. After being blocked with the goat serum, the slices were incubated with anti‐ZHX2 polyclonal antibody (Proteintech, Wuhan, China) at a dilution of 1 : 200 in distilled water overnight at 4 °C. Then, the expression of ZHX2 was detected using a SABC test kit (BOSTER, Wuhan, China).

The immunohistochemical results were assessed by two independent pathologists. The ZHX2 protein expression score was derived from the staining intensity and the proportion of stained cells. The staining intensity was graded as: 0 (−), 1 (+), 2 (++) and 3 (+++). The stained area was scored as: 1 (< 25%); 2 (26–50%); 3 (51–75%) and 4 (> 75%). An immunoreactivity score ≥ 4 was considered to represent high expression.

### Cell culture

The human GC cell lines SGC‐7901, MGC‐803, AGS, MKN‐45 and BGC‐823 were purchased from the Shanghai Cell Bank at the Chinese Academy of Sciences (Shanghai, China). The immortalized normal gastric mucosal epithelial cell line (GES‐1) was obtained from the Tumor Laboratory at the First Affiliated Hospital of Chongqing Medical University. Cells were cultured in 1640 medium (Gibco, Gaithersburg, MD, USA) and DMEM (HyClone, Logan, UT, USA). The culture medium was supplemented with 10% fetal bovine serum (BIOIND, Kibbutz Beit‐Haemek, Israel).

### Real‐time quantitative PCR

The total RNA of cells was extracted using TRIzol (Takara, Shiga, Japan) and transcribed into cDNA using a Primer Script RT Kit (Takara). SYBR Green (Takara) was used for a quantitative real‐time quantitative PCR (qRT‐PCR). The primers were ordered from GeneCopoeia (Rockville, MD, USA). Relative gene expression level was calculated via $2^{‐\Delta \Delta C_{T}}$.

### Cell transfection

ZHX2 overexpression and its control lentivirus were obtained from HanBio (Shanghai, China). AGS cells cultured in six‐well plates were infected with overexpressing lentivirus particles or scrambled control clone with polybrene (5 µg·mL^−1^; Sigma, St Louis, MO, USA). Stable cells overexpressing ZHX2 were selected using puromycin (Roche, Indianapolis, IN, USA) after transfection. The small interfering RNA used to interfere with the expression of ZHX2 and the respective negative control were synthesized by Ribio (Shanghai, China) and BGC‐823 cells were transfected with small interfering RNA using Lipo2000 (Invitrogen, Carlsbad, CA, USA).

### Cell counting kit‐8 (CCK‐8) and colony formation

The transfected cells were cultured in 96‐well plates at a density of 10^3^ cells per well, and cell viability was measured at the indicated times, 24, 48, 72 and 96 h after incubating with CCK‐8 solution (Beyotime, Shanghai, China). Cells after transfection were cultured in six‐well plates at a density of 500 cells per well and incubated for 10 days. After incubation, cells were fixed and dyed by 0.5% crystal violet. Clone numbers were counted for analysis.

### Cell apoptosis

The apoptosis of cells after transfection was tested by flow cytometry and an annexin V‐propidium iodide staining kit (Solarbio, Beijing, China) was applied. After transfection, cells were planted on six‐well plates for 48 h. Cells were digested and stained with 5 μL of annexin V and propidium iodide after incubation. The cell apoptosis rate was analyzed using a CytoFlex flow cytometer (Beckman Coulter, Brea, CA, USA).

### Wound healing and transwell invasion assays

Cells were planted on the six‐well plates after transfection and cultured in an incubator. A 200‐μL pipette tip was equipped to scratch across the cells. Cells were imaged after 0 and 24 h and the image‐pro plus (https://www.mediacy.com/imageproplus) was used to measure the distance of cell migration.

Matrigel (Corning, NY, USA) was used to coat the upper chambers. After transfection, cells were resuspended in 200 mL culture medium and cultured in the top chambers (Corning Inc., Corning, NY, USA) and 600 mL of complete culture medium was added into the basolateral chamber. Cells under the membrane were fixed and then dyed using 5% crystal violet.

### Western blotting

Cells were added with lysis buffer and total protein was extracted. Equal amounts of protein samples were added to SDS/PAGE for conducting electrophoresis. Next, after being blocked for 15 min with QuickBlock solution (Beyotime), the membranes with proteins were incubated with primary antibodies against ZHX2 (Proteintech) and β‐actin (Proteintech, Wuhan, China). Then, the membranes were incubated with the secondary antibodies and the proteins in the membranes were visualized by ECL after washing with TBST. β‐actin was used as a reference.

### Gene set enrichment analysis

Gene set enrichment analysis (GSEA) (https://www.gsea‐msigdb.org) was used to detect the significantly enriched pathways related to ZHX2 using whole‐genome expression profiling in GC. RNA‐sequencing data were obtained from the TCGA portal and the gene sets were divided into high and low two groups depending on their relationship with the median expression level of ZHX2. One thousand times permutations were conducted to screen the enriched pathways between the two groups. Nominal *P* < 0.01 with a false discovery rate *q* value < 0.25 was considered as statistically significant.

### ESTIMATE

ESTIMATE (https://bioinformatics.mdanderson.org/public‐software/estimate) comprises a comprehensive resource for analyzing stromal cell and immune cell infiltration in tumors. ESTIMATE used the TCGA gene expression profiles to infer the immune score, stromal score and estimate score, which was a combination of the aforementioned two scores and indicates tumor purity. The immune score represented immune cell infiltration, whereas the stromal score reflected stromal cell infiltration.

### Statistical analysis

The data were analyzed using spss, version 22.0 (IBM Corp., Armonk, NY, USA). The correlation between ZHX2 expression and clinicopathological characteristics was analyzed by chi‐squared analysis. Kaplan–Meier and Cox analyses were applied for survival analysis. The significance of different groups was analyzed by a *t*‐test or one‐way analysis of variance. *P* < 0.05 was considered statistically significant.

## Results

### A high mRNA expression level of ZHX2 in GC predicts poor prognosis

**Fig. 1:**
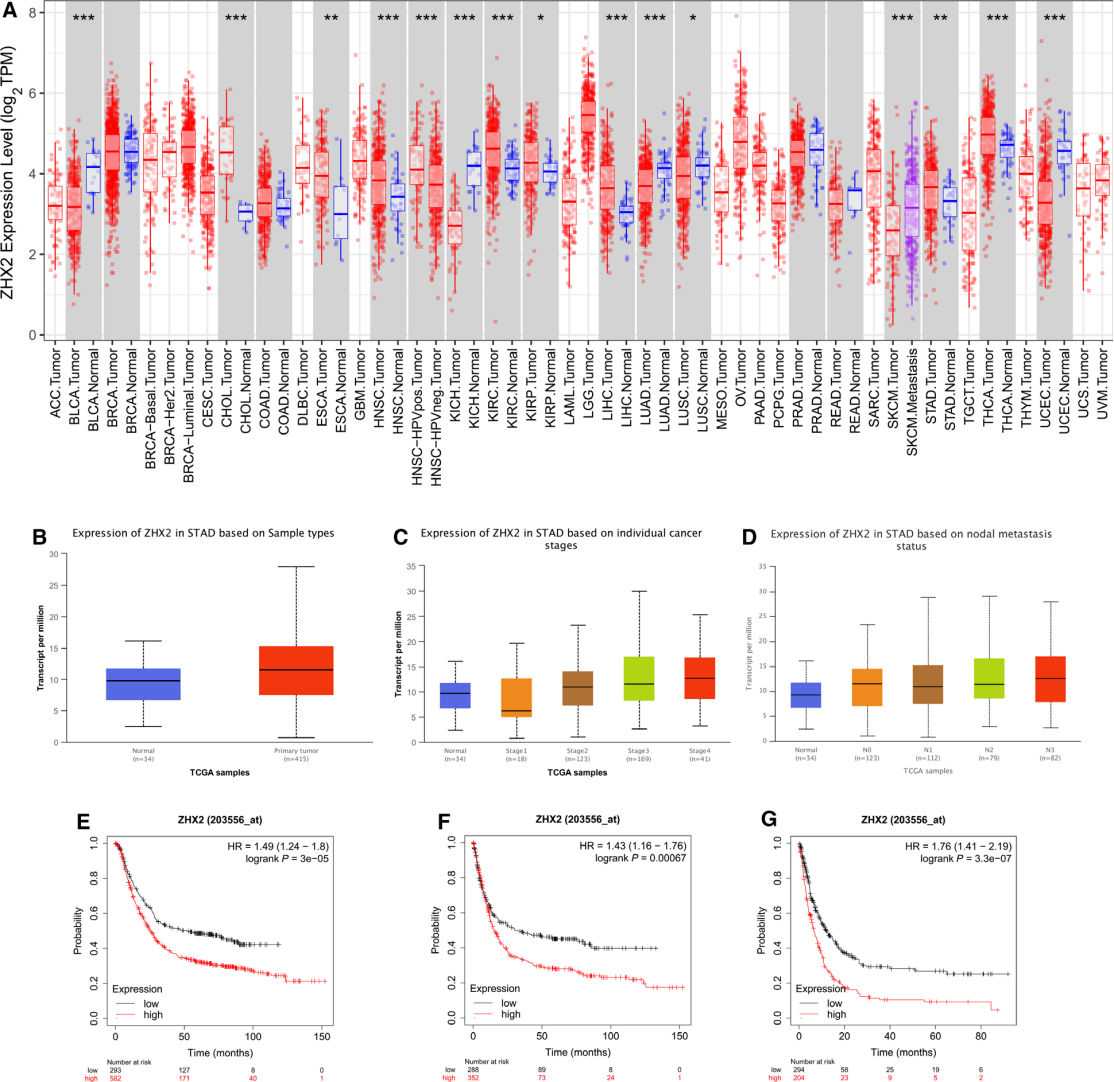
A high mRNA expression level of ZHX2 in GC is correlated with clinicopathological characteristics and poor survival. (A) The mRNA expression level of ZHX2 in different cancers compared to normal tissues from TCGA databases analyzed by TIMER. (B) The mRNA expression level of ZHX2 in GC analyzed by UCLCAN. (C, D) The mRNA expression level of ZHX2 in different clinicopathological characteristics, including stage (C) and nodal metastasis (D), in GC. (E–G) Kaplan–Meier curve for the OS (E), FP (F) and PPS (G) of patients with GC from Kaplan–Meier plotter databases.

The tumor is heterogeneous and the difference in ZHX2 expression in diverse tissues might provide new insights into the function and mechanism of ZHX2. We utilized the TIMER database to evaluate ZHX2 mRNA levels in diverse cancers. The expression level of ZHX2 was increased in cholangiocarcinoma, esophageal carcinoma, head and neck squamous cell carcinoma, head and neck squamous cell carcinoma‐HPV pos, kidney renal clear cell carcinoma, kidney renal papillary cell carcinoma, liver hepatocellular carcinoma, skin cutaneous melanoma‐metastasis, stomach adenocarcinoma and thyroid carcinoma, whereas there was a decrease of ZHX2 expression levels in bladder urothelial carcinoma, kidney chromophobe, lung adenocarcinoma, lung squamous cell carcinoma and uterine corpus endometrial carcinoma (Fig. 1A). To further confirm the mRNA expression level of ZHX2 in GC, the UCLCAN database was applied. ZHX2 was significantly highly expressed in GC tissues (*n* = 415) compared to gastric tissues (*n* = 34) (Fig. 1B). Moreover, we also analyzed the correlation of ZHX2 with clinicopathological characteristics. The mRNA expression level of ZHX2 between normal tissues and stage 1 GC tissues demonstrated no significant difference, whereas an increased ZHX2 mRNA level was observed in GC tissues from stage 2 to stage 4 (Fig. 1C). Furthermore, the mRNA expression level of ZHX2 was obviously upregulated in N0, N1, N2 and N3 groups compared to the normal node group (Fig. 1D). Additionally, we also utilized Kaplan–Meier Plotter to investigate the role of ZHX2 with respect to the prognosis of GC patients. A high mRNA expression level of ZHX2 was correlated with a shorter OS, FP and PPS (Fig. 1E–G). In brief, the above results indicated that the high mRNA level of ZHX2 predicted a poor prognosis in GC.

### The protein expression level of ZHX2 is upregulated and associated with the clinicopathological characteristics in GC

**Fig. 2:**
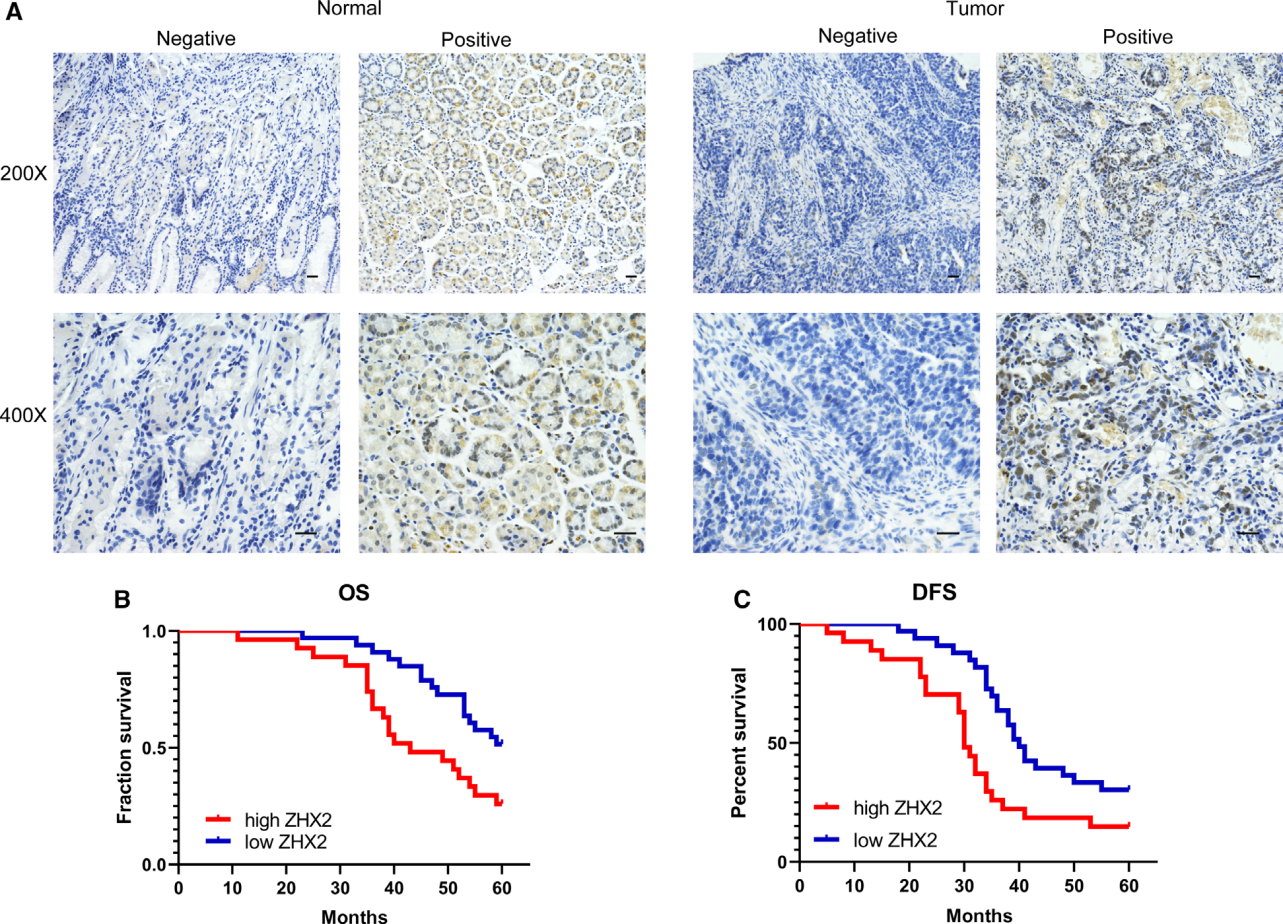
A high protein expression of ZHX2 in GC is correlated with poor survival in clinical samples. (A) Representative image of negative and positive ZHX2 expression in normal tissues and GC tissues using IHC. (B, C) Kaplan–Meier survival curves for OS (B) of patients and DFS (C) of patients according to ZHX2 expression. Scale bar = 100 μm.

To further confirm the role of ZHX2 in GC, IHC was then used to analyze the protein expression level of ZHX2 in 60 paired GC tissues and corresponding normal tissues from our hospital. The ZHX2 positive staining was primarily observed in the cell nucleus. Representative images of ZHX2 expression in GC tissues and adjacent normal tissues are shown in Fig. 2A. ZHX2 showed high expression levels in 27 (45%) and low or negative expression in 33 (55%) GC tissues, whereas ZHX2 was highly expressed in 11 (18.3%) and lowly (or not) expressed in 49 (81.7%) adjacent normal tissues. The expression of ZHX2 in gastric cancer tissues was significantly higher than that in adjacent normal tissues (Table 1).

**Table 1:** The expression of ZHX2 in gastric cancer tissues and adjacent normal tissues.

Group	*n*	ZHX2 expression		*χ* ^2^	*P*
		Low	High		
Gastric cancer	60	33	27	9.859	0.003
Adjacent normal	60	49	11		

Correlations between the protein expression level of ZHX2 and the clinicopathological characteristics are shown in Table 2. The expression of ZHX2 was not associated with the degree of differentiation, tumor size or lymph metastasis but was related to advanced T stage and TNM stage. No significant association was identified with the expression of ZHX2 and the other clinical characteristics, such as gender and age. Collectively, these results indicated that the protein level of ZHX2 was significantly upregulated in GC and correlated with advanced T stage and TNM stage.

**Table 2:** Association between ZHX2 expression and clinical pathological characteristics in patients with gastric cancer.

	*n*	ZHX2 expression		*χ* ^2^	*P*
		Low	High		
Total	60	33	27		
Gender					
Male	38	19	19	1.047	0.420
Female	22	14	8		
Age					
< 60	27	12	15	2.210	0.193
≥ 60	33	21	12		
Tumor size					
< 3 cm	27	14	13	0.197	0.795
≥ 3 cm	33	19	14		
Differentiation					
Well	9	3	6	2.008	0.276
Moderate/poor	51	30	21		
T stage					
T1/T2	13	11	2	5.881	0.025
T3/T4	47	22	25		
TNM stage					
I/II	26	19	7	6.058	0.019
III/IV	34	14	20		
Lymph node metastasis					
No	22	16	6	4.411	0.059
Yes	38	17	21		

### The protein level of ZHX2 is associated with overall survival and disease‐free survival in GC patients

Kaplan–Meier analysis was applied to evaluate the correlation between the protein expression level of ZHX2 and prognosis in GC patients. Patients with high ZHX2 expression had a shorter OS than those with low ZHX2 expression (Fig. 2B). In the univariate analysis, ZHX2 expression, T stage and TNM stage were associated with the OS. In the multivariate analysis, ZHX2 expression and TNM stage were also associated with the OS (Table 3). Patients with low ZHX2 expression also had a longer disease‐free survival (DFS) than those with high ZHX2 expression (Fig. 2C). In univariate analysis, ZHX2 expression, lymph metastasis and TNM stage were associated with DFS. Multivariate analysis was also used to detect the protein expression level of ZHX2 and the related parameters. ZHX2 expression and TNM stage were associated with DFS and indicated that ZHX2 could function as a potential prognostic factor in GC (Table 4).

**Table 3:** Univariate and multivariate Cox proportional hazards models of the expression of ZHX2 and OS for patients with gastric cancer. CI, confidence interval.

Variable	Univariate analysis			Multivariate analysis		
	Hazard ratio	95% CI	*P*	Hazard ratio	95% CI	*P*
Gender	1.225	0.626–2.395	0.557			
ZHX2 expression	2.262	1.168–4.382	0.016	1.991	1.017–3.896	0.044
Age	1.200	0.618–2.330	0.590			
Tumor size	1.384	0.713–2.687	0.337			
Differentiation	0.837	0.348–2.018	0.693			
T stage	4.300	1.316–14.052	0.016			
Lymph node metastasis	2.053	0.964–4.372	0.062			
TNM stage	2.587	1.244–5.378	0.011	2.319	1.104–4.870	0.026

**Table 4:** Univariate and multivariate Cox proportional hazards models of the expression of ZHX2 and DFS for patients with gastric cancer. CI, confidence interval.

Variable	Univariate analysis			Multivariate analysis		
	Hazard ratio	95% CI	*P*	Hazard ratio	95% CI	*P*
Gender	0.816	0.440–1.513	0.518			
ZHX2 expression	2.155	1.200–3.870	0.010	1.982	1.096–3.584	0.024
Age	1.115	0.624–1.994	0.713			
Tumor size	1.347	0.752–2.415	0.317			
Differentiation	0.837	0.566–1.238	0.373			
T stage	2.116	0.981–4.562	0.056			
Lymph node metastasis	2.366	1.232–4.543	0.010			
TNM stage	2.436	1.314–4.514	0.005	2.283	1.222–4.267	0.010

Taken together, these above data suggested that ZHX2 could function as a biomarker for the prognostic evaluation of GC.

### ZHX2 promotes the proliferation and inhibits the apoptosis of GC cells

**Fig. 3:**
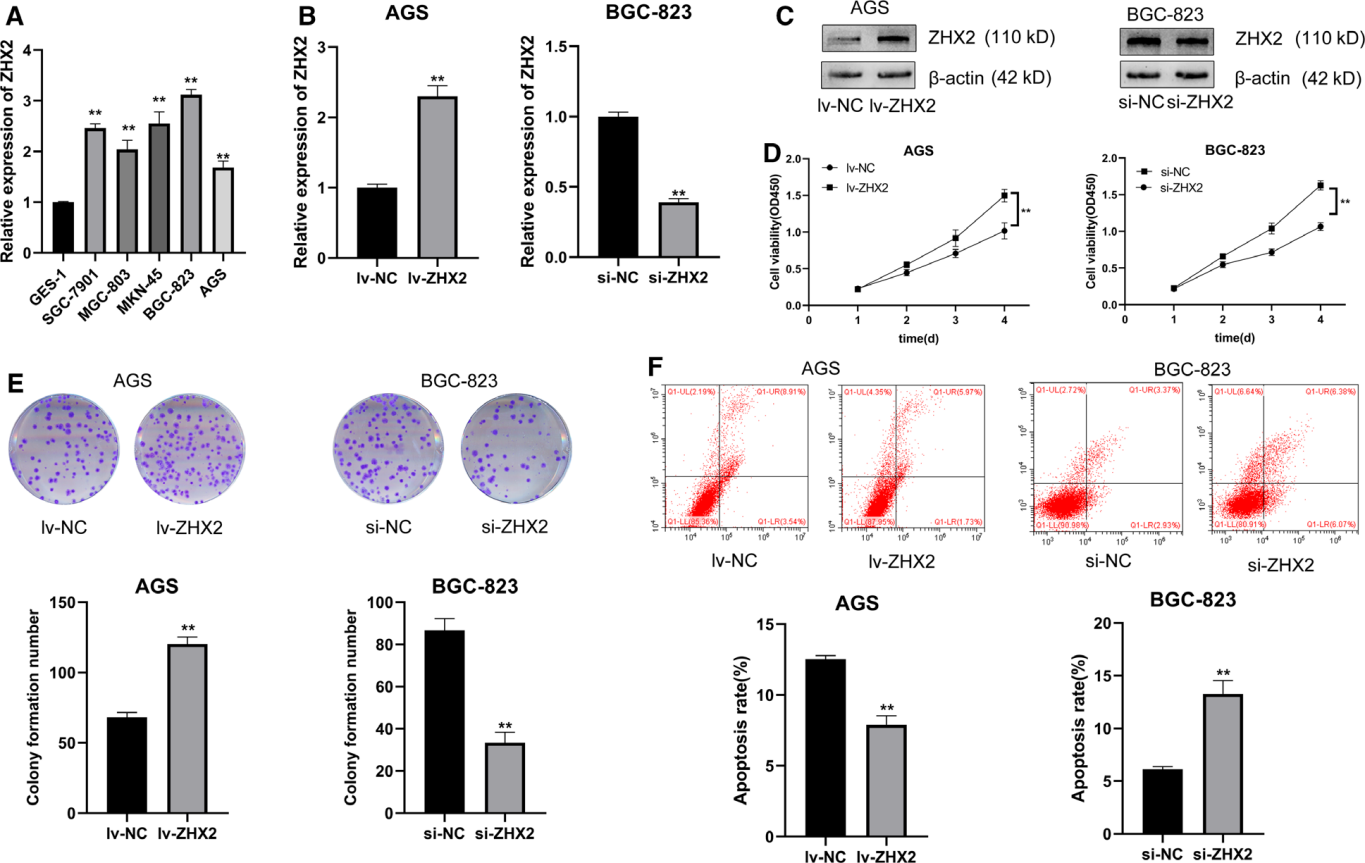
ZHX2 promotes the proliferation and inhibits the apoptosis of GC cells. (A) Expression levels of ZHX2 in gastric cancer cell lines, including SGC‐7901, AGS, BGC‐823, MGC‐803, MKN‐45 and GES‐1, were determined by qRT‐PCR. Differences between groups were compared using one‐way analysis of variance. (B, C) ZHX2 overexpression and silencing effects were examined by qRT‐PCR (B) and western blotting (C) in AGS and BGC‐823 cell lines. (D) A CCK‐8 assay was performed to measure cell viability after transfection. (E) A colony formation assay was used to test cell proliferation of AGS and BGC‐823 cells. (F) Cell apoptosis was analyzed by flow cytometry. Differences between two groups were compared using a Student’s *t*‐test. Data represent the mean ± SD of three separate experiments. **P < 0.001.

**Fig. 4:**
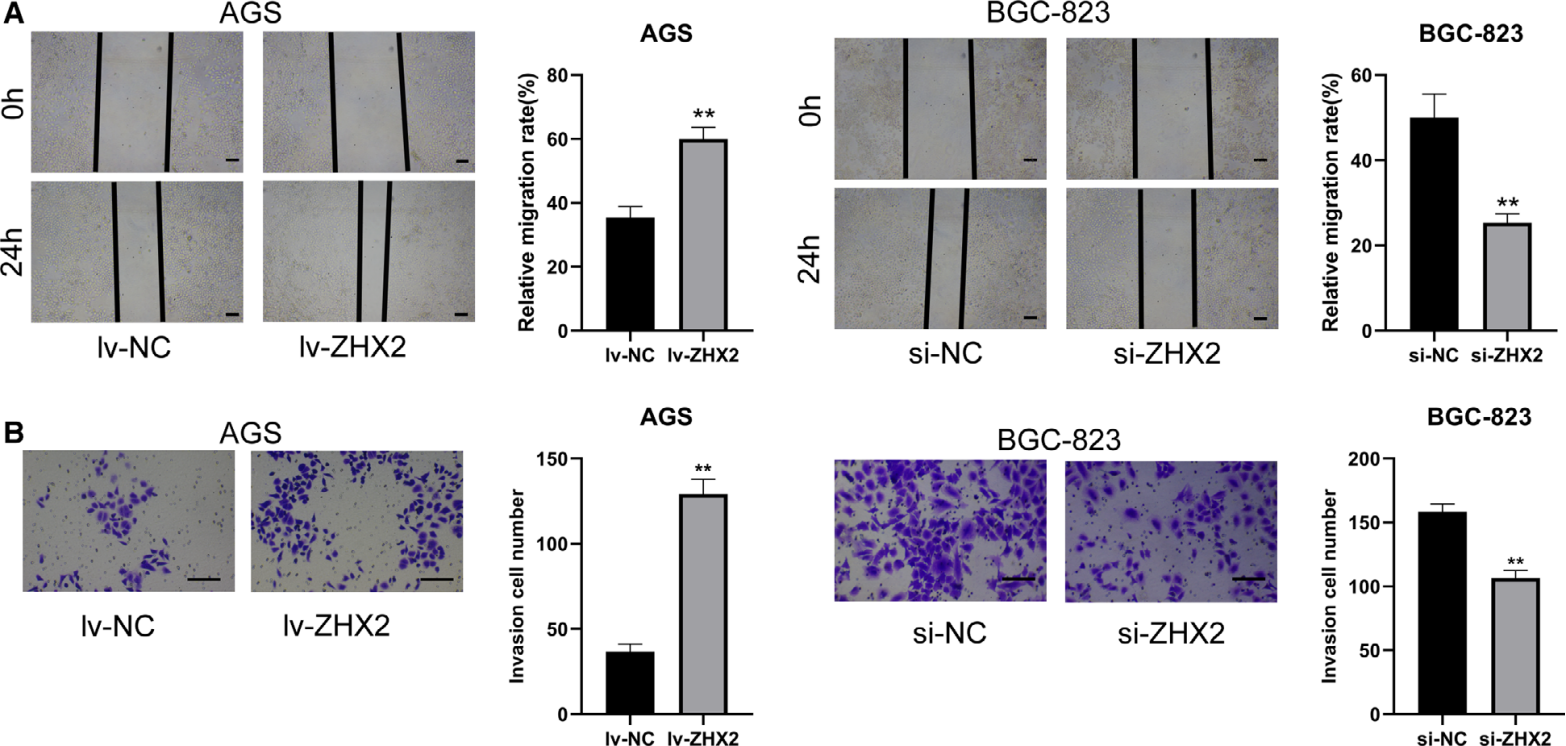
ZHX2 promotes cell migration and invasion. (A) A wound healing assay was used to detect cell migration of AGS and BGC‐823 cells. Scale bar = 200 μm. (B) A transwell invasion assay was applied to detect the effect of overexpressing and silencing ZHX2 with respect to the invasion of AGS and BGC‐823 cells. Scale bar = 200 μm. Differences between two groups were compared using a Student's *t*‐test. Data represent the mean ± SD of three separate experiments. **P < 0.001.

To explore the role of ZHX2 *in vitro*, we tested the expression of ZHX2 in GC cell lines and found that ZHX2 was significantly upregulated in gastric cancer cell lines (Fig. 3A). Given that ZHX2 was highly expressed in GC tissues and associated with a poor prognosis, we further investigated its role by overexpressing ZHX2 in AGS cells and silencing ZHX2 in BGC‐823 cells. The mRNA and protein expression levels of ZHX2 were increased in AGS cells transfected with overexpressing lentivirus, and silencing ZHX2 in BGC‐823 cells decreased the expression level of ZHX2 (Fig. 3B,C). Next, we conducted functional experiments. A CCK‐8 assay indicated that cell viability was enhanced in AGS cells by overexpressing ZHX2 and inhibited by silencing ZHX2 in BGC‐823 cells (Fig. 3D). The data from the colony formation assay also showed that overexpressing ZHX2 increased the proliferation in AGS cells, whereas silencing ZHX2 inhibited the proliferation in BGC‐823 cells (Fig. 3E). Flow cytometry revealed that overexpressing ZHX2 strikingly inhibited the apoptosis in AGS cells, whereas silencing ZHX2 increased the percentage of apoptosis in BGC‐823 cells (Fig. 3F). Wound healing and transwell invasion assays were conducted to evaluate the effect of ZHX2 on cell migration and invasion. As shown in Fig. 4A, the migration capabilities of AGS cells were significantly enhanced by overexpression of ZHX2 and decreased after silencing ZHX2 in BGC‐823 cells. A transwell invasion assay demonstrated that overexpressing ZHX2 promoted the invasion ability of AGS cells, whereas silencing ZHX2 inhibited the invasion ability of BGC‐823 cells (Fig. 4B).

### GSEA identifies ZHX2 related signalling pathways in GC

**Fig. 5:**
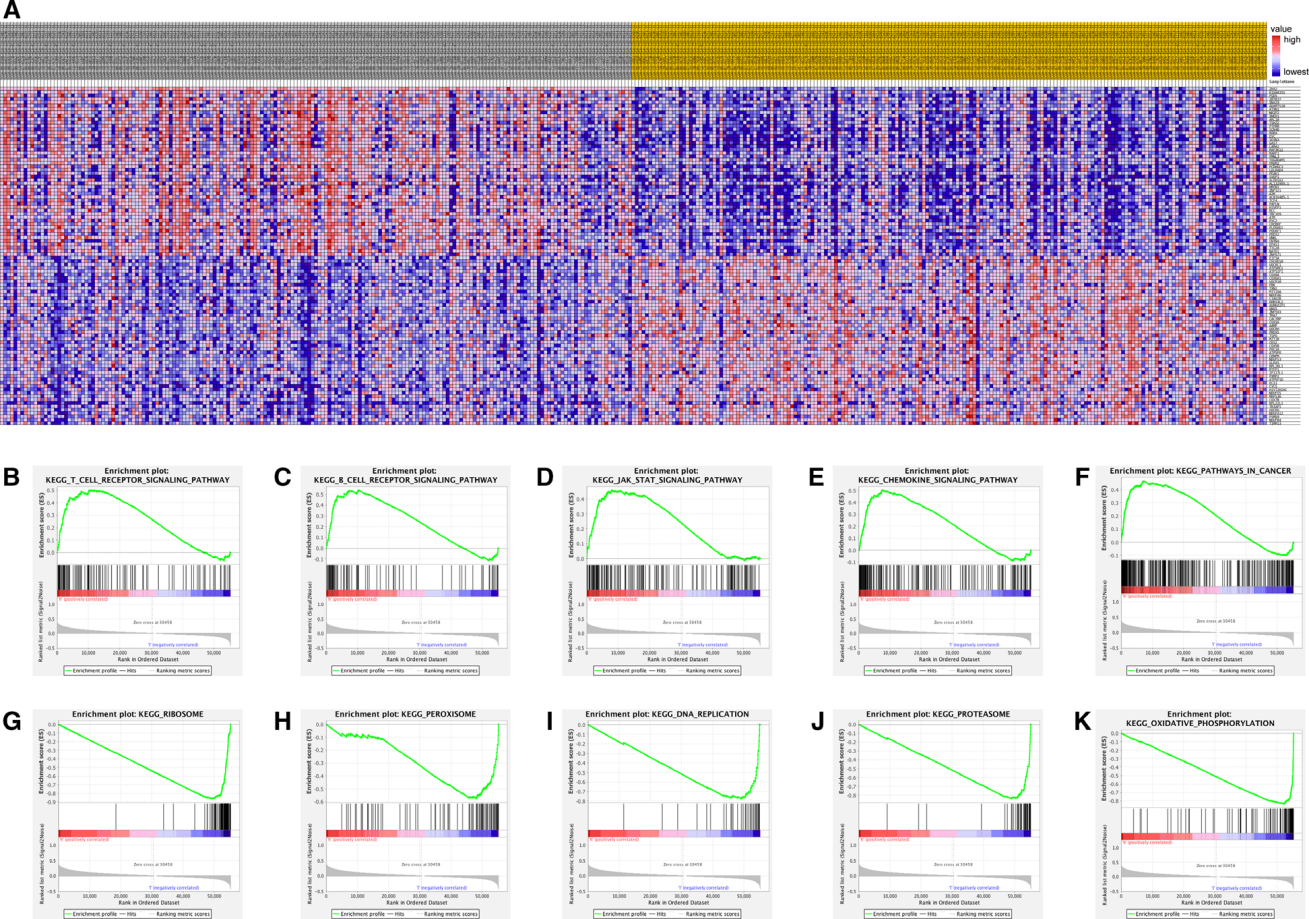
GESA of ZHX2 from TCGA GC samples. (A) Heat map of the top 50 features for each phenotype in high‐ and low‐ ZHX2 groups. (B–F) The five notable Kyoto Encyclopedia of Genes and Genomes (KEGG) pathways (https://www.genome.jp/kegg) in the high ZHX2 expression group. (B) T cell receptor signaling pathway. (C) B cell receptor signaling pathway. (D) JAK‐STAT signaling pathway. (E) Chemokine signaling pathway. (F) Pathways in cancer. (G–K) The five notable KEGG pathways in the low ZHX2 expression group. (G) Ribosomes. (H) Peroxisomes. (I) DNA replication. (J) Proteasomes. (K) Oxidative phosphorylation.

We conducted GSEA to identify signalling pathways related to ZHX2 in GC (Fig. 5A). High expression of ZHX2 was remarkedly correlated with various pathways, including the T cell receptor signalling pathway, B cell receptor signalling pathway, chemokine signalling pathway, JAK‐STAT signalling pathway and pathways in cancer, and so on (Fig. 5B–F), whereas low expression of ZHX2 was associated with ribosomes, peroxisomes, DNA replication, proteasomes and oxidative phosphorylation, and so on (Fig. 5G–K).

**Fig. 6:**
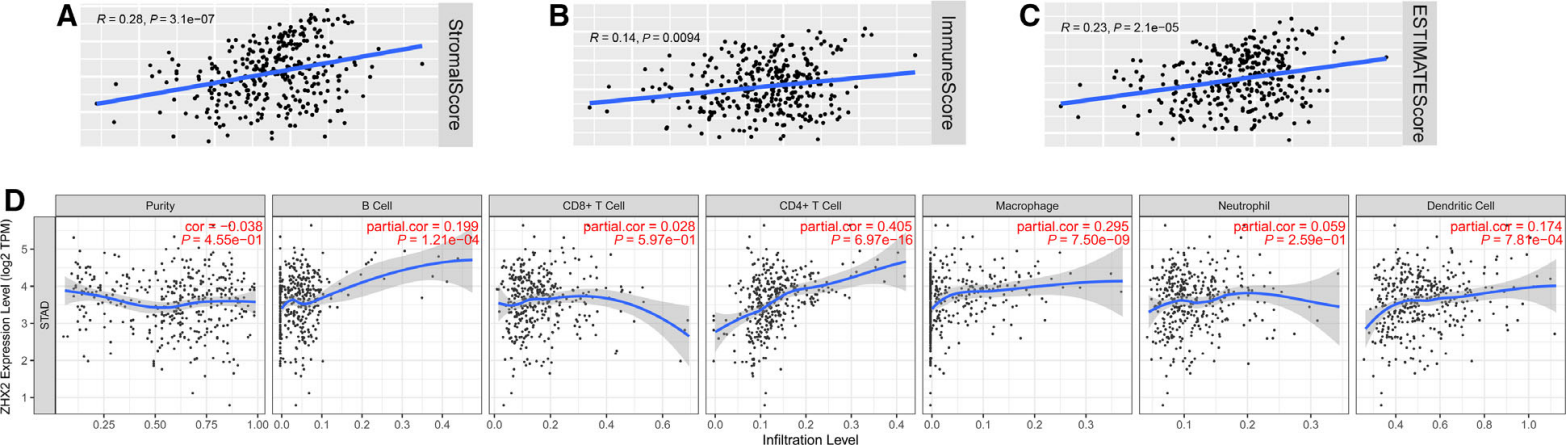
High expression of ZHX2 is correlated with immune infiltration in GC. (A–C) Correlation of ZHX2 with immune infiltration analyzed by ESTIMATE. (A) Stromal, (B) immune and (C) ESTIMATE scores. (D) Correlation of ZHX2 with immune infiltration cells analyzed by TIMER, including tumor purity, B cells, CD8^+^ T cells, CD4^+^ T cells, macrophaged, neutrophils and dendritic cells.

Given that the immune infiltration in tumor microenvironment could effectively predict the prognosis of cancer patients [13] and the above pathways from the GSEA results of the high ZHX2 group were also closely correlated with immune related pathways, we evaluated the correlation between the expression level of ZHX2 and immune infiltration. ESTIMATE indicated that a high ZHX2 expression level lead to increased immune, stromal and estimate scores (Fig. 6A–C), suggesting that ZHX2 was correlated with immune infiltration. Moreover, a detailed analysis of immune infiltrating cells using TIMER demonstrated that ZHX2 was positively associated with B cells, CD4^+^ T cells, macrophages and dendritic cells (Fig. 6D). Furthermore, we also analyzed the relationship between ZHX2 and the marker genes of immune infiltration cells, including CD8^+^ T cells, B cells, T cells (general), monocytes, tumor‐associated macrophages, M1 macrophages, M2 macrophages, natural killer cells, neutrophils and dendritic cells. Additionally, diverse T cell subgroups, such as Th1, Th2, Tfh, Th17, Tregs and exhausted T cells, were also analyzed (Table 5). There was a strong correlation between ZHX2 and STAT5B, which was a marker gene of Tregs. Other Tregs related marker genes also showed a significantly positive correlation with ZHX2, indicating that ZHX2 might active Tregs. Notably, ZHX2 expression was positively correlated with several marker genes of T helper cells, including Th1 cells, Th2 cells, Tfh and Th17 cells, indicating a regulatory role of ZHX2 on T cell function. Moreover, moderate correlations could be observed between ZHX2 expression and the marker genes of dendritic cell, suggesting that ZHX2 might regulate the immune infiltration of dendritic cells. Taken together, the above results suggested that ZHX2 was correlated with immune infiltration cells.

**Table 5:** Correlation analysis between ZHX2 and marker genes of immune infiltration cells in TIMER. TAM, tumor‐associated macrophage; Th, T helper cell; Tfh, follicular helper T cell; Treg, regulatory T cell; Cor, *r* value of Spearman's correlation; none, correlation without adjustment; purity, correlation adjusted by purity.

Description	Gene markers	None		Purity	
		Cor	*P* value	Cor	*P* value
CD8^+^ T cells	CD8A	0.116	1.77 × 10^–2^	0.095	6.47 × 10^–2^
	CD8B	0.139	*	0.122	1.74 × 10^–2^
T cells (general)	CD3D	0.105	2.23 × 10^–2^	0.078	1.28 × 10^–1^
	CD3E	0.154	*	0.125	1.47 × 10^–2^
	CD2	0.142	*	0.121	1.85 × 10^–2^
B cells	CD19	0.274	***	0.265	***
	CD79A	0.212	***	0.192	**
Monocytes	CD86	0.167	**	0.15	*
	CD115 (CSF1R)	0.288	***	0.275	***
TAM	CCL2	0.158	*	0.135	*
	CD68	0.127	*	0.114	2.64 × 10^–2^
	IL10	0.241	***	0.235	***
M1 macrophages	INOS (NOS2)	0.028	5.75 × 10^–1^	0.021	6.79 × 10^–1^
	IRF5	0.335	***	0.323	***
	COX2 (PTGS2)	0.19	**	0.184	**
M2 macrophages	CD163	0.279	***	0.274	***
	VSIG4	0.179	**	0.178	**
	MS4A4A	0.221	***	0.209	***
Neutrophils	CD11b (ITGAM)	0.335	***	0.324	***
	CD66b (CEACAM8)	0.142	*	0.157	*
	CCR7	0.352	***	0.337	***
Natural killer cells	KIR2DL1	–0.036	4.61 × 10^–1^	–0.049	3.44 × 10^–1^
	KIR2DL3	–0.023	6.33 × 10^–1^	–0.028	5.93 × 10^–1^
	KIR2DL4	–0.086	8.16 × 10^–2^	–0.087	9.16 × 10^–2^
	KIR3DL1	0.009	8.54 × 10^–1^	0.024	6.48 × 10^–1^
	KIR3DL2	0.006	9.07 × 10^–1^	0.012	8.09 × 10^–1^
	KIR3DL3	–0.058	2.36 × 10^–1^	–0.038	4.63 × 10^–1^
	KIR2DS4	–0.046	3.53 × 10^–1^	–0.045	3.77 × 10^–1^
Dendritic cells	HLA‐DPB1	0.131	*	0.105	4.14 × 10^–2^
	HLA‐DQB1	0.092	6.19 × 10^–2^	0.07	1.75 × 10^–1^
	HLA‐DRA	0.103	3.67 × 10^–2^	0.08	1.22 × 10^–1^
	HLA‐DPA1	0.112	2.25 × 10^–3^	0.089	8.5 × 10^–2^
	BDCA‐1 (CD1C)	0.357	***	0.342	***
	BDCA‐4 (NRP1)	0.444	***	0.432	***
	CD11c (ITGAX)	0.27	***	0.267	***
Th1	T‐bet (TBX21)	0.123	1.20 × 10^–2^	0.112	2.98 × 10^–2^
	STAT4	0.294	***	0.29	***
	STAT1	0.091	6.51 × 10^–2^	0.094	6.82 × 10^–2^
	IFN‐γ (IFNG)	0.054	2.69 × 10^–1^	–0.054	2.94 × 10^–1^
	TNF‐α (TNF)	0.11	2.46 × 10^–2^	0.085	1.90 × 10^–1^
Th2	GATA3	0.275	***	0.271	***
	STAT6	0.349	***	0.349	***
	STAT5A	0.394	***	0.395	***
	IL13	0.013	7.85 × 10^–1^	0.012	8.14 × 10^–1^
Tfh	BCL6	0.456	***	0.456	***
	IL21	0.029	5.5 × 10^–1^	0.023	6.53 × 10^–1^
Th17	STAT3	0.453	***	0.455	***
	IL17A	–0.106	3.05 × 10^–2^	–0.107	3.66 × 10^–2^
Treg	FOXP3	0.188	**	0.174	**
	CCR8	0.243	***	0.238	***
	STAT5B	0.597	***	0.591	***
	TGFβ (TGFB1)	0.354	***	0.349	***
T cell exhaustion	PD‐1 (PDCD1)	0.164	**	0.154	*
	CTLA4	0.132	*	0.123	1.67 × 10^–2^
	LAG3	–0.029	5.60 × 10^–1^	–0.046	3.69 × 10^–1^
	TIM‐3 (HAVCR2)	0.159	*	0.146	*
	GZMB	–0.134	*	–0.153	*

* *P* < 0.01

** *P* < 0.001

*** *P* < 0.0001

## Discussion

The incidence of GC is increasing and its cancer‐related burden is high in many countries, where it creates enormous consequences for society and economics [14]. Even though advances in digestive endoscopy, serum tumor markers and radiological related technology have improved the early diagnosis and treatment options, the prognosis of GC remains poor [15]. GC is heterogeneous and is influenced by geographic location and individual susceptibility. The application of genomics has revealed more insights into genetic changes between tumors in different locations and at different times [16]. Genomics analyses can also provide new clues for elucidating the underlying mechanism of GC tumorigenesis and progression.

ZHX2, a nonspecific transcription suppressor factor, is a member of the ZHX family that contains two zinc fingers and four HDs. *ZHX2* is located at 8q24.13 and has a total cDNA length of 4500 bp, which codes an 837 amino acid protein containing a proline‐rich region between HD1 and HD2 [17]. ZHX2 can form homologous dimers by binding itself and form a heterodimer by binding with ZHX1 or ZHX3 at the HD1 domain [18]. In hepatocellular cancer, the expression level of ZHX2 in the nucleus was significantly decreased and correlated with tumor differentiation [19]. Perincheri *et al*. [20] reported that ZHX2 could inhibit the expression of AFP in adult mice, a finding in accordance with hepatocellular cancer patients. ZHX2 has been reported to regulate the replication of hepatitis B virus, decrease hepatitis B viral load and inhibit hepatocellular cancer progression [21]. ZHX2 could also downregulate the expression of multiple drug resistance gene at the transcriptional level to enhance the susceptibilities of tumor cells to chemotherapy drugs in hepatocellular cancer [22] and myeloma [23]; however, the deficiency of von Hippel–Lindau could upregulate the expression of ZHX2 and result in increased binding between ZHX2 and H2K4me3, which activates the nuclear factor‐kappa B pathway in renal clear cell carcinoma [12]. Taken together, these reports reveal that ZHX2 is closely correlated with the tumorigenesis in various cancers and might play different roles in different cancers.

In the present study, we found that the mRNA and protein level of ZHX2 were both significantly higher in GC and the upregulation of ZHX2 predicted the poor survival of patients with GC from both TCGA databases and our samples. This finding is in line with the oncogenic role identified for ZHX2 in renal clear cell cancer, although it is the opposite of that in hepatocellular cancer. Next, the functional experiments demonstrated that ZHX2 could promote the cell proliferation, migration, invasion and impeded the apoptosis in GC, whereas silencing ZHX2 had the opposite effect. Furthermore, GSEA analysis was conducted to detect the underlying mechanism of ZHX2 in GC and a subset of the pathways related with immune response were identified. ZHX2 achieved high immune, stromal and estimate scores for ZHX2 via the ESTIMATE algorithm, indicating a correlation of ZHX2 with immune infiltration. Then, ZHX2 was demonstrated to be correlated with the infiltration of CD4^+^ T cells, macrophages, B cells and dendritic cells by TIMER. Finally, to further confirm the relationship between ZHX2 and immune infiltrating cells, we analyzed the connection between ZHX2 and marker genes of immune infiltration cells and revealed that ZHX2 was also positively correlated with the marker genes of T helper cells (Th1, Th2, Tfh, Th17 and Tregs) and dendritic cells. Th1 [24], Th2 [25] and Th17 [26] in GC also predicted poor prognosis, and these results indicated that ZHX2 might regulate the T cell function to affect tumorigenesis in GC.

Tregs, a subset of immunosuppressive CD4^+^T cells, could influence the anti‐tumor immune responses and were correlated with a poor prognosis in cancers [27]. Tregs were upregulated in various cancer tissues and the peripheral blood of cancer patients including GC [28]. In the present study, a high expression of ZHX2 was correlated with Tregs marker genes (STAT5B and TGF‐β). Recent studies have demonstrated that blocking TGF‐β signaling in CD4^+^T cells remodeled the tumor microenvironment and restrained cancer progression [29] and depletion of transforming growth factor‐β receptor 2 (TGFBR2) in CD4^+^ T cells promoted cancer progression [30]. Whether ZHX2 could remodel the tumor microenvironment in a TGF‐β dependent pathway in CD4^+^ T cells requires further exploration.

Dendritic cells, an immune component of the tumor microenvironment, could shape the immune response by presenting antigens and providing signals for T cell activation and differentiation. Dendritic cells were reported to increase Tregs and reduce CD8^+^ T cell cytotoxicity and promote tumor metastasis in breast cancer [31]. Regarding GC, dendritic cells were upregulated in patients with GC and predicted a poor prognosis [32]. The correlation of ZHX2 and dendritic cells also confirmed the regulation of ZHX2 with respect to the immune infiltration of dendritic cells. These results indicated that ZHX2 was correlated with diverse immune infiltration cells in GC and thus might affect the progression of GC. However, further experiments are needed to explore the underlying mechanism of ZHX2 with respect to regulating immune infiltration.

## Conclusions

The present study confirms that ZHX2 is highly expressed in GC at both mRNA and protein expression levels. Upregulation of ZHX2 is correlated with clinicopathological characteristics and predicts poor survival in GC. ZHX2 could promote the proliferation, migration and invasion and inhibit the apoptosis of GC cells. The immune infiltration might account for the oncogenic role of ZHX2 in GC. These data might offer insights into the development of novel diagnostics and therapeutics for GC.

## Conflict of interests

The authors declare that they have no conflicts of interest.

## Author contributions

AC designed and conducted the experiments and also wrote the manuscript. XG and XD analyzed the data. ZW supervised the research and revised the manuscript. All authors read and approved the final version of the manuscript submitted for publication.

## Data Availability

The raw data are available from the corresponding author upon reasonable request.

## References

[1] Bray F , Ferlay J , Soerjomataram I , Siegel RL , Torre LA and Jemal A (2018) Global cancer statistics 2018: GLOBOCAN estimates of incidence and mortality worldwide for 36 cancers in 185 countries. CA Cancer J Clin 68, 394–424. 30207593 10.3322/caac.21492

[2] Fitzmaurice C , Dicker D , Pain A , Hamavid H , Moradi‐Lakeh M , MacIntyre MF , Allen C , Hansen G , Woodbrook R , Wolfe C *et al*. (2015) The global burden of cancer 2013. JAMA Oncol 1, 505–527. 26181261 10.1001/jamaoncol.2015.0735 PMC4500822

[3] Ajani JA , Lee J , Sano T , Janjigian YY , Fan D and Song S (2017) Gastric adenocarcinoma. Nat Rev Dis Primers 3, 17036. 28569272 10.1038/nrdp.2017.36

[4] Smyth EC , Nilsson M , Grabsch HI , van Grieken NC and Lordick F (2020) Gastric cancer. Lancet 396, 635–648. 32861308 10.1016/S0140-6736(20)31288-5

[5] Ward ZJ , Scott AM , Hricak H , Abdel‐Wahab M , Paez D , Lette MM , Vargas HA , Kingham TP and Atun R (2020) Estimating the impact of treatment and imaging modalities on 5‐year net survival of 11 cancers in 200 countries: a simulation‐based analysis. Lancet Oncol 21, 1077–1088. 32758462 10.1016/S1470-2045(20)30317-X PMC8020599

[6] Li C , Chen W , Jiang F , Simino J , Srinivasan SR , Berenson GS and Mei H (2015) Genetic association and gene‐smoking interaction study of carotid intima‐media thickness at five GWAS‐indicated genes: the Bogalusa Heart Study. Gene 562, 226–231. 25746325 10.1016/j.gene.2015.02.078

[7] Tian X , Wang Y , Li S , Yue W and Tian H (2020) ZHX2 inhibits proliferation and promotes apoptosis of human lung cancer cells through targeting p38MAPK pathway. Cancer Biomark 27, 75–84. 31683461 10.3233/CBM-190514 PMC12662273

[8] Kwon RJ , Kim YH , Jeong DC , Han ME , Kim JY , Liu L , Jung JS and Oh SO (2017) Expression and prognostic significance of zinc fingers and homeoboxes family members in renal cell carcinoma. PLoS One 12, e0171036. 28152006 10.1371/journal.pone.0171036 PMC5289508

[9] Olsson M , Lindahl G and Ruoslahti E (1977) Genetic control of alpha‐fetoprotein synthesis in the mouse. J Exp Med 145, 819–827. 67170 10.1084/jem.145.4.819 PMC2180631

[10] Shen H , Luan F , Liu H , Gao L , Liang X , Zhang L , Sun W and Ma C (2008) ZHX2 is a repressor of alpha‐fetoprotein expression in human hepatoma cell lines. J Cell Mol Med 12, 2772–2780. 18194454 10.1111/j.1582-4934.2008.00233.x PMC3828890

[11] Zhang J , Wu T , Simon J , Takada M , Saito R , Fan C , Liu XD , Jonasch E , Xie L , Chen X *et al*. (2018) VHL substrate transcription factor ZHX2 as an oncogenic driver in clear cell renal cell carcinoma. Science 361, 290–295. 30026228 10.1126/science.aap8411 PMC6154478

[12] You Y , Bai F , Li H , Ma Y , Yao L , Hu J and Tian Y (2020) Prognostic value and therapeutic implications of ZHX family member expression in human gastric cancer. Am J Transl Res 12, 3376–3388. 32774706 PMC7407748

[13] Chang WJ , Du Y , Zhao X , Ma LY and Cao GW (2014) Inflammation‐related factors predicting prognosis of gastric cancer. World J Gastroenterol 20, 4586–4596. 24782611 10.3748/wjg.v20.i16.4586 PMC4000495

[14] Chen W , Zheng R , Baade PD , Zhang S , Zeng H , Bray F , Jemal A , Yu XQ and He J (2016) Cancer statistics in China, 2015. CA Cancer J Clin 66, 115–132. 26808342 10.3322/caac.21338

[15] Wei W , Zeng H , Zheng R , Zhang S , An L , Chen R , Wang S , Sun K , Matsuda T , Bray F *et al*. (2020) Cancer registration in China and its role in cancer prevention and control. Lancet Oncol 21, e342–e349. 32615118 10.1016/S1470-2045(20)30073-5

[16] Lim B , Kim JH , Kim M and Kim SY (2016) Genomic and epigenomic heterogeneity in molecular subtypes of gastric cancer. World J Gastroenterol 22, 1190–1201. 26811657 10.3748/wjg.v22.i3.1190 PMC4716030

[17] Kawata H , Yamada K , Shou Z , Mizutani T , Yazawa T , Yoshino M , Sekiguchi T , Kajitani T and Miyamoto K (2003) Zinc‐fingers and homeoboxes (ZHX) 2, a novel member of the ZHX family, functions as a transcriptional repressor. Biochem J 373, 747–757. 12741956 10.1042/BJ20030171 PMC1223552

[18] Kawata H , Yamada K , Shou Z , Mizutani T and Miyamoto K (2003) The mouse zinc‐fingers and homeoboxes (ZHX) family; ZHX2 forms a heterodimer with ZHX3. Gene 323, 133–140. 14659886 10.1016/j.gene.2003.09.013

[19] Song X , Tan S , Wu Z , Xu L , Wang Z , Xu Y , Wang T , Gao C , Gong Y , Liang X *et al*. (2018) HBV suppresses ZHX2 expression to promote proliferation of HCC through miR‐155 activation. Int J Cancer 143, 3120–3130. 29752719 10.1002/ijc.31595

[20] Perincheri S , Dingle RW , Peterson ML and Spear BT (2005) Hereditary persistence of alpha‐fetoprotein and H19 expression in liver of BALB/cJ mice is due to a retrovirus insertion in the Zhx2 gene. Proc Natl Acad Sci USA 102, 396–401. 15626755 10.1073/pnas.0408555102 PMC544306

[21] Xu L , Wu Z , Tan S , Wang Z , Lin Q , Li X , Song X , Liu Y , Song Y , Zhang J *et al*. (2018) Tumor suppressor ZHX2 restricts hepatitis B virus replication via epigenetic and non‐epigenetic manners. Antiviral Res 153, 114–123. 29580980 10.1016/j.antiviral.2018.03.008

[22] Ma H , Yue X , Gao L , Liang X , Yan W , Zhang Z , Shan H , Zhang H , Spear BT and Ma C (2015) ZHX2 enhances the cytotoxicity of chemotherapeutic drugs in liver tumor cells by repressing MDR1 via interfering with NF‐YA. Oncotarget 6, 1049–1063. 25473899 10.18632/oncotarget.2832 PMC4359216

[23] Legartova S , Harnicarova‐Horakova A , Bartova E , Hajek R , Pour L and Kozubek S (2010) Expression of RAN, ZHX‐2, and CHC1L genes in multiple myeloma patients and in myeloma cell lines treated with HDAC and Dnmts inhibitors. Neoplasma 57, 482–487. 20568903 10.4149/neo_2010_05_482

[24] Zhang H , Yue R , Zhao P , Yu X , Li J , Ma G , Tang J , Zhang L , Feng L , Sun L *et al*. (2017) Proinflammatory follicular helper T cells promote immunoglobulin G secretion, suppress regulatory B cell development, and correlate with worse clinical outcomes in gastric cancer. Tumour Biol 39, 1010428317705747. 28631561 10.1177/1010428317705747

[25] Wei M , Shen D , Mulmi Shrestha S , Liu J , Zhang J and Yin Y (2018) The progress of T cell immunity related to prognosis in gastric cancer. Biomed Res Int 2018, 3201940. 29682534 10.1155/2018/3201940 PMC5848132

[26] Li Q , Li Q , Chen J , Liu Y , Zhao X , Tan B , Ai J , Zhang Z , Song J and Shan B (2013) Prevalence of Th17 and Treg cells in gastric cancer patients and its correlation with clinical parameters. Oncol Rep 30, 1215–1222. 23807713 10.3892/or.2013.2570

[27] Dangaj D , Bruand M , Grimm AJ , Ronet C , Barras D , Duttagupta PA , Lanitis E , Duraiswamy J , Tanyi JL , Benencia F *et al*. (2019) Cooperation between constitutive and inducible chemokines enables T cell engraftment and immune attack in solid tumors. Cancer Cell 35, 885–900.e10. 31185212 10.1016/j.ccell.2019.05.004 PMC6961655

[28] Ichihara F , Kono K , Takahashi A , Kawaida H , Sugai H and Fujii H (2003) Increased populations of regulatory T cells in peripheral blood and tumor‐infiltrating lymphocytes in patients with gastric and esophageal cancers. Clin Cancer Res 9, 4404–4408. 14555512

[29] Li S , Liu M , Do MH , Chou C , Stamatiades EG , Nixon BG , Shi W , Zhang X , Li P , Gao S *et al*. (2020) Cancer immunotherapy via targeted TGF‐beta signalling blockade in TH cells. Nature 587, 121–125. 33087933 10.1038/s41586-020-2850-3 PMC8353603

[30] Liu M , Kuo F , Capistrano KJ , Kang D , Nixon BG , Shi W , Chou C , Do MH , Stamatiades EG , Gao S *et al*. (2020) TGF‐β suppresses type 2 immunity to cancer. Nature 587, 115–120. 33087928 10.1038/s41586-020-2836-1 PMC8347705

[31] Sawant A , Hensel JA , Chanda D , Harris BA , Siegal GP , Maheshwari A and Ponnazhagan S (2012) Depletion of plasmacytoid dendritic cells inhibits tumor growth and prevents bone metastasis of breast cancer cells. J Immunol 189, 4258–4265. 23018462 10.4049/jimmunol.1101855 PMC3531993

[32] Liu W , Zhao J , Li Q , Wang Q , Zhou Y and Tong Z (2018) Gastric cancer patients have elevated plasmacytoid and CD1c(+) dendritic cells in the peripheral blood. Oncol Lett 15, 5087–5092. 29552142 10.3892/ol.2018.7990 PMC5840537

